# Vulnerability through the Eyes of People Attended by a Portuguese Community-Based Association: A Thematic Analysis

**DOI:** 10.3390/healthcare10101819

**Published:** 2022-09-21

**Authors:** Carlos Laranjeira, Inês Piaça, Henrique Vinagre, Ana Rita Vaz, Sofia Ferreira, Lisete Cordeiro, Ana Querido

**Affiliations:** 1 School of Health Sciences, Polytechnic of Leiria, Campus 2, Morro do Lena, Alto do Vieiro, Apartado 4137, 2411-901 Leiria, Portugal; 2 Centre for Innovative Care and Health Technology (ciTechCare), Polytechnic of Leiria, Campus 5, Rua de Santo André—66–68, 2410-541 Leiria, Portugal; 3 Research in Education and Community Intervention (RECI I&D), Piaget Institute, 3515-776 Viseu, Portugal; 4 InPulsar (Associação para o Desenvolvimento Comunitário), Rua José Gonçalves LT 55—LJ 3 PISO-1, 2410-121 Leiria, Portugal; 5 Center for Health Technology and Services Research (CINTESIS), NursID, University of Porto, 4200-450 Porto, Portugal

**Keywords:** vulnerability, qualitative study, social risk, community intervention

## Abstract

Vulnerability is associated with the individual’s social and biological conditions, but also the conditions of their enveloping environment and society, leading to terms such as vulnerable populations or risk groups. This study aimed to give a voice to people with experiences of vulnerability and explore their perspectives, using a descriptive qualitative design. Purportedly vulnerable adults were recruited and interviewed with semi-structured questions on vulnerability. Data were organized, using WebQDA software, and submitted to thematic content analysis, as proposed by Clark and Braun, which generated a thematic tree. The study included six men and six women with a mean age of 43.8 [SD = 14.17] years old. Thematic analysis generated three themes: (1) Conceptions about vulnerability, (2) Barriers imposed by vulnerability, and (3) Strategies for dealing with vulnerability. The results highlight that vulnerability is a highly dynamic process of openness to circumstances that influence individual outcomes. However, there is a lack of conceptual clarity. Although being vulnerable is perceived as something negative, we need to transform the social mindset, because vulnerability also has the potential to change priorities in life for the better.

## 1. Introduction

Vulnerability is a key concept to health and social welfare and is commonly applied in academic research and policy formulation. Indeed, vulnerable people are “individuals or groups who require support with social, health, or economic problems” [1] (p. 2). Although there is no precise definition of vulnerability or vulnerable persons, the literature suggests common indicators of vulnerability and/or vulnerable groups [2]. Typically, it refers to a lack of physical, psychological, and social well-being, and hence the risk of lagging behind or becoming socially isolated from society [3,4]. Other indicators commonly stressed in the literature include: “(a) accumulation of problems or limitations (multi-complex problems); (b) feelings of powerlessness and distrust; (c) disrupted communication; (d) limited or no access to resources; (e) marginality; (f) imbalance in burden and capacity; (g) dependency situation; and (h) low self-esteem” [2] (p. 2). The ongoing COVID-19 pandemic has raised the vulnerability of many people, with several health implications. The disease and its repercussions—including dietary deficits, physical and mental health issues (potentially decreasing awareness of distinct hazards), and more limited access to healthcare services—were factors exacerbated by the pandemic [3,5].

According to Herring [6], there are two main conceptualizations of vulnerability: the dominant perspective in the literature classifies some persons or groups as vulnerable, whereas the alternative perspective emphasizes universal human vulnerability [6,7]. The latter view is uncommon in the literature, appearing largely in a psychological or even therapeutical context [8], but is highly promising, as stressed by Martha Fineman. According to her, universal vulnerability bears with it the impending or ever-present risk of damage, pain, and tragedy [1,9]. However, Fineman does not dispute that certain people or groups are more exposed to vulnerability than others [1]. Vulnerability is both universal and specific. As a multifaceted experience, it can take a variety of forms, accumulate and be passed down through generations. Fineman also used vulnerability as an interpersonal notion, referring to relational interactions, particularly those between people and society [9]. Fineman [9] concentrates on the social processes that generate vulnerability, as well as the duty of the state and its institutions/systems to decrease their risks and effects. Indeed, vulnerability analysis must consider individual circumstances, social processes, society, and its institutions [1,10]. Thus, exploring the vulnerability experiences offers a richer and wider perspective of vulnerability.

### 1.1. Theoretical Framework

Vulnerability is a key concept in health and social care disparities [11]. Based on this assumption, Flaskerud and Winslow [12] developed a conceptual model that characterises vulnerability as a complex interaction between susceptibility, risk, resource availability and health status. From a social justice stance, the authors propose that the interplay between these concepts may guide research and support evidence-based policy. With their knowledge and experience, institutions and vulnerable groups can help develop care strategies to mitigate the effects of vulnerability [13].

Based on the ecological systems theory model created by Bronfenbrenner [14], addressing vulnerability necessitates identifying its causes and drivers, as well as clarifying its expanding concept. In this sense, several factors influencing vulnerability in an individual’s family, community and society at large cannot be taken out of this context [14,15]. As the risk of being impacted by exogenous events, vulnerability can be influenced by economic variables (either linked to welfare or development), socio-political factors, or environmental variables. These sources of vulnerability correlate with dimensions commonly mentioned in the sustainable development agenda. Vulnerability appears as the polar opposite of sustainability in these three domains [16]; it is a danger to sustainability.

### 1.2. Research Problem

Portugal has been indicated as one of the most vulnerable countries in the European Union [17]. In 2019, 9.8% of the Portuguese population was in persistent poverty, with 6% in poverty over the previous four years; unemployment or family disruption were the most common causes [18]. Thus, groups that are considered vulnerable in both economic and social terms [19]. Moreover, living in extreme poverty contributes to the perpetuation of risky behaviours, resulting in a greater risk of social and health problems.

The “exclusion of vulnerable groups from codesign processes may result in a failure to challenge dominant constructions of health and social care that may unintentionally reinforce oppression and existing inequities” [20] (p. 285). Growing evidence shows that research co-production may do more than just help put research findings into practice; it can also boost the possibility that public services are appropriately adapted to the requirements of the communities they serve [20,21]. In contrast, evidence shows conceptual confusion surrounding the meaning and consequences of the concept of vulnerability [22], requiring additional research among people with first-hand experiences of being vulnerable.

To the best of our knowledge, there is a dearth of qualitative research on vulnerability from the viewpoint of the vulnerable. To address this gap, using a participatory bottom-up approach, this study aims to explore the perceptions and experiences of vulnerability from the perspective of vulnerable people and identify strategies they used to reduce vulnerability.

## 2. Materials and Methods

### 2.1. Study Design

This study used a qualitative descriptive design. Through a naturalistic inquiry, this study aimed to comprehend the specifics of vulnerability in the natural context and based on the perspective of those involved [2,23]. The COnsolidated criteria for REporting Qualitative research (COREQ) checklist were followed [24].

### 2.2. Study Setting, Participants and Recruitment

Gathering data with people in a vulnerable state can be challenging [25]. Access to the target population often needs cooperation with local social services. Therefore, our study scenario was a non-governmental organization (InPulsar-Association for Community Development) located in the region of Leiria (Portugal), whose mission is to contribute to the social and economic inclusion of vulnerable populations. Their intervention process is based on the participatory action-research methodology: a network intervention based on observation, reflection, planning and evaluation of the various actors. The aim is to achieve the emancipation and autonomy of beneficiaries and their inclusion in the local community. Training and empowerment are also encouraged.

This organization was selected because it provided the research team with access to the vulnerable population. Potential participants were approached and informed about the study by one institutional facilitator (L.C.), with previous knowledge and experience working with vulnerable people.

The sample size was based on purposeful sampling because we used a qualitative exploratory research technique. The following criteria were applied in the selection process: (1) participants who fulfilled the current criteria and/or characteristics of “vulnerable people”; (2) adults (at least 18 years old); (3) understand the Portuguese language and have reflective capacity; and (4) participants with a personal sense of vulnerability or the experience of being vulnerable.

Gender and age distribution diversity were also considered. Eligible responders undergoing medical or psychological therapy were excluded from the study, to avoid any detrimental influence on the person’s treatment. People with cognitive disabilities or weak communication abilities were also excluded.

### 2.3. Data Collection

The study was conducted in April 2022 and employed a semi-structured interview guide to perform the in-depth interviews. The authors developed the interview guide based on the previous literature [2]. Interviews began with getting to know the participant and then exploring the meaning of vulnerability for the individual, the forms it assumes, how it is viewed, and when in life it was experienced. Interviews ended with discussing insights into potential measures to lessen perceived vulnerability, in order to grasp the perceived vulnerability on a personal and interactional level.

All interviews took place in a private room of InPulsar where distractions were minimal. Each interview was digitally recorded and lasted approximately 30 min (varying between 20 and 50 min). No repeat interviews were carried out.

### 2.4. Data Analysis

Transcripts of interview recordings were imported into WebQDA (Universidade de Aveiro, Aveiro, Portugal), a qualitative software program, for thematic content analysis according to the Braun and Clarke [26] guidelines. First, data familiarization was accomplished by reading all transcripts. Second, pertinent information was grouped into understandable codes. Third, codes were categorized into probable vulnerability-related topics. Fourth, thematic validity was confirmed by reviewing all codes and the complete data set. Fifth, topics were defined and named, producing a final thematic tree. Finally, a literature review was used to help write the report [27].

Coding involved two (co)researchers: the lead researcher (C.L.) coded all transcripts, which were then independently co-coded by the co-researcher with a scientific background and competence in data processing (A.Q.). When differences or disagreements arose during co-coding, the coders reflected until consensus was obtained. To reach a final decision, a third researcher (L.C.) was consulted. Selected transcript quotes from Portuguese were translated, italicized, and inserted into the following report.

### 2.5. Study Rigour

The interviews were planned and performed by the research team, and all interviewers (final year nursing students) were supervised by leading co-researchers (C.L. and A.Q.), who have an insider’s viewpoint and practice knowledge of vulnerability. To guarantee reliability and validity, the interviewers underwent interview training and reflexive sessions, including discussion about the interview guide, and, during the data collection period, participated in regular feedback sessions based on recorded interviews.

Findings were peer-reviewed by co-authors (investigator triangulation) and resulted in the revision of theme and sub-themes names to rightfully capture the essence of the findings. We also kept an audit trail, which included field notes, coded transcripts, and comments and revisions from group coding meetings, following the best practices in qualitative research [28].

### 2.6. Ethics

The study was conducted after approval by the Ethics Committee of the Polytechnic of Leiria (CE/IPLEIRIA/02/2022). All participants provided written informed consent, and all sociodemographic data, interview recordings, transcriptions, and files including IDs were saved and password-protected, accessible only to members of the study team. Respondents received no compensation for participating in the study.

## 3. Results

### 3.1. Sample Description

The 12 respondents varied in age from 24 to 67 years old (mean = 49; SD = 14.17), with six males and six women (Table 1). The manifestations of vulnerability reported by participants included being homeless, being a migrant, having an infectious disease (e.g., HIV and hepatitis), being drug dependent, living socioeconomic difficulties (unemployment), and experiencing a process of loss and grief. Three respondents worked, while the remainder received social benefits. Three participants attributed the cause of their homelessness and their inability to find suitable accommodation to a lack of family support, substance abuse, and other mental health concerns.

Some respondents reported having a mental or physical health problem, or both. Depression and anxiety were the most often reported mental health disorders. Participants recognized the significance that substance abuse had in their mental health, but also highlighted how drugs and alcohol provided short-term respite. Use of alcohol and drugs serves to block traumatic experiences or deal with painful childhood memories, usually including abuse, loss, and bereavement. Physical health difficulties such as hepatitis C, physical impairments, and HIV were quite common. As greater emphasis was given to their mental health, housing, and drug abuse, these were rarely regarded as a priority.

### 3.2. Qualitative Findings

**Figure 1:**
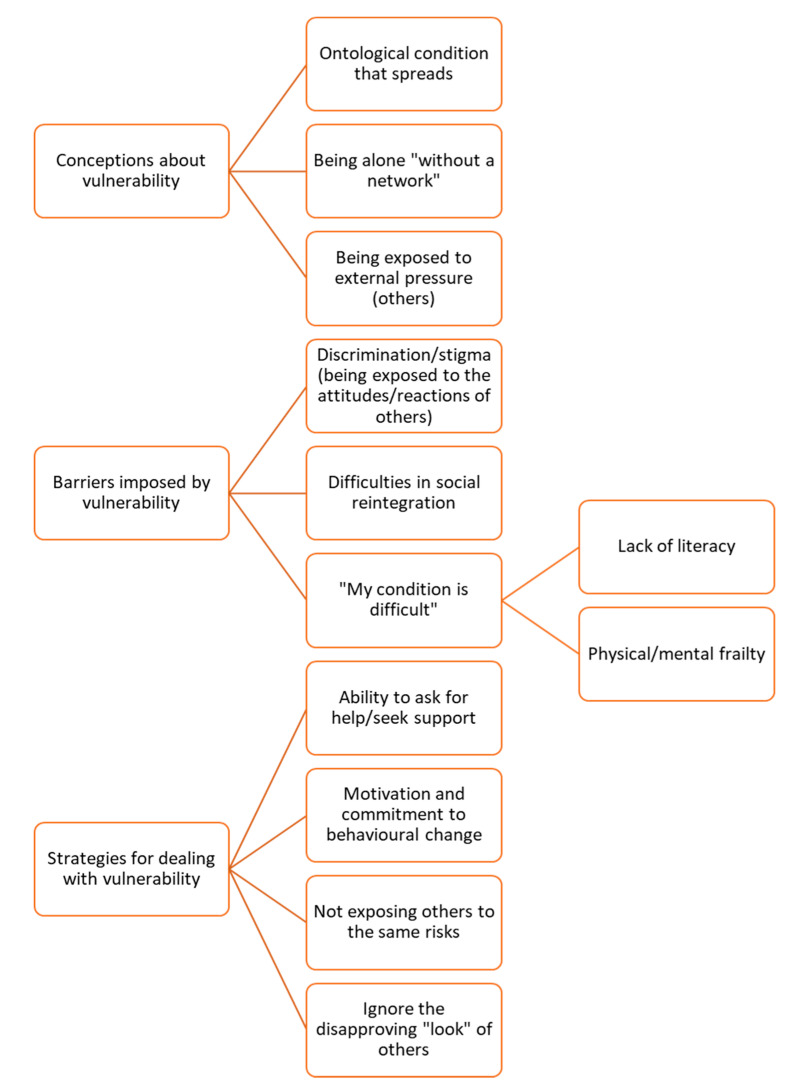
Thematic map of themes and subthemes.

The data from our interviews can be summarized in terms of three major themes: (1) Conceptions about vulnerability, (2) Barriers imposed by vulnerability, and (3) Strategies for dealing with vulnerability. Three subthemes were identified within the first theme: ontology condition that spreads, being alone “without network” and being exposed to external pressure (others). In the second theme, there were also three subthemes: discrimination/stigma, difficulties in social reintegration, and “my condition is difficult”. Lastly, in the third theme, we found four subthemes: the ability to ask for help/seek support, motivation, and commitment to behavioural change, not exposing others to the same risks, and ignoring the disapproving look of others. All themes and subthemes are represented in Figure 1 and are considered crucial in determining participants’ understanding of vulnerability. Of course, some elements of participants’ comprehension are related to more than one theme. However, this ought to be seen as a good interpretation of perceptions and attitudes generally, which are never made up of distinct ideas but rather relate to one another in various ways. Each theme is discussed below, and meaningful quotes were included to support the findings.

#### 3.2.1. Conceptions about Vulnerability

An individual’s notion, belief, or sense of anything known, experienced, or imagined is referred to as a conception. There are several meanings attributed to vulnerability, derived from each participant’s life context. The first theme describes vulnerability as an ontological condition that spreads, that is, the person sees vulnerability as something intrinsic to their life and that runs through their existence:

*P1: “It’s part of a period of life that I lived, when I was living on the street, that’s when I felt this effect the most (…) Yes, it’s a snowball, and it affects our whole life (…) I lived on the street because the money I received from the unemployment fund was not enough to pay for a room and now due to drug consumption I continue to live on the street and spend all my money on drugs”.*

*P10: “During my childhood my father beat me because yes… so I felt vulnerable, because I couldn’t do anything, I thought why was he doing that to me, why? And he felt vulnerable because he couldn’t do anything. Now he still feels vulnerable because I can’t stop consuming”.*

From the analysis of the discourses, it is evident that vulnerability is not a unique and isolated phenomenon, but rather a condition that can accompany the subject throughout life, interfering with their functioning.

Another frequent theme in this dimension was being alone “without a network”. Four participants addressed how loneliness interferes with the way they perceive and experience vulnerability:

*P5: “The truth is that I need a friend to be able to talk, so I don’t get to this point of keeping everything, and then when it explodes it’s like this (…), after my father died, after 6 months my brother left home and my mother worked at night (…) I was always alone, so I’ve been used to being alone for a long time (…) It’s been very difficult, I don’t know what to do with my life, what I can do myself…”*

*P7: “I have no support from anyone, not even the family (…) anyone! I only have the support of my son, nothing else, I stopped talking to them, because I am of a nature that they do not admit. I did so much for them, but they don’t recognize…”*

*P9: “I prefer to be alone in my corner, because I have no one to help me”*

*P10: “Alone (…) I don’t have anyone, my father died, my wife died, I mean I don’t have anyone else”*

Lack of support and loneliness are conditions that promote situations of vulnerability, since participants do not have anyone to share their feelings and concerns and feel isolated. Participants mentioned failures in the quality of the relationship networks, then there are less relationships than desired or retribution is not proportional.

Finally, the theme of being exposed to external pressure (others) arose associated with the adoption of risky behaviours, beyond their personal will. In this sense, P4 accepted what was asked, suggested or instilled, as a way of pleasing others.

*P4: “Yes, it’s not just for me, it’s because of my environment, I think people are like that, a little weak and they always go after each other; and it is easy, for example, to find someone who has never smoked anything; and they say then don’t you want to try it? Or they see me smoking and ask to try it. If they didn’t call me I wouldn’t go. I never have money, but when I do, I think straight away, of course I do (…) Dealers call me every day, write “there are big scenes in the neighbourhood, you get 50€ for two”, “you take 50€ and you get a bonus”, scenes like that …”*

Vulnerability resulting from social pressure seems to arise from an imbalance between the socio-emotional system and the cognitive control system. If on the one hand there is a need for acceptance, on the other hand, there is a lack of personal and social skills (e.g., assertiveness) to say “no” to psychoactive substances.

#### 3.2.2. Barriers Imposed by Vulnerability

Some participants mentioned barriers associated with their vulnerability, referring to the importance of identifying vulnerability early in the hope of avoiding its perpetuation. The main topic addressed in this dimension was discrimination/stigma, i.e., the exposure of participants to stereotypes and negative attitudes of others, namely their own family and society in general.

*P4: “Yes, I think that people, at least my family, think I’m a drug addict and look at me as a drug addict, and that’s why I don’t get a job, because I’m not all hot to go to work”.*

*P6:” Oops, when I was on the street, they looked at me differently”.*

*P7: “Yes, I do, the recriminatory look of people”.*

*P8: “They look from the side (…) some people, not all”.*

*P9: “There were people who discriminated against me, others who tried to help me and I didn’t want to; sometimes not accepting help makes things more difficult!”*

*P10: “I say I have leishmaniasis and people look at me in a different way! And they ask, can you catch Filipe? If you get that scared, how much more if I said I have HIV, it’s not…”*

P9 underlined that, despite some prejudice, other people were available to help, but refusing that help perpetuated isolation, a characteristic of the most vulnerable. Some participants also pointed out that stigma affected childhood and adolescence, as well as the search for employment in adulthood.

*P3:” I’ve been criticized many times and they said you’re like this because you want to, and you don’t have a job because you don’t want to. But it’s not quite like that, I was made fun of at school for not wearing designer clothes, or for being chubby, or because my mother doesn’t have a profession that is said to be worthy. My mother, always did everything to not miss us with anything…”*

*P2: “I have missing teeth and when I look for a job, there are people who look at me and say “we just want younger people”. My image doesn’t help me, I know, but what can I do…”*

Akin to discrimination/stigma, participants also referred difficulty in social reintegration.

*P1: “At the moment, what I feel is difficulty in integrating myself into society again, it seems that I am dependent on everything (…) I even wanted to attend professional training, but they said that it would be difficult to enter”.*

*P7: “At my age, it’s so hard to find a job! I’ve been looking for an alternative for months and nothing…”*

Social reintegration assumes the character of reconstructing losses, and its objective is to enable people to fully exercise their right to citizenship. However, difficulties in social reintegration are attributed to deficient living conditions, namely in the search for employment and/or education/training.

The third theme—my condition is difficult—is subdivided into two sub-themes. The first sub-theme refers to the lack of literacy, which portrays how a lack of education became an obstacle in the lives of the participants. In this regard, P7 mentioned being sad for not having the necessary resources to secure a job.

*P7: “(…) I have no studies. I would like to know, even more, read and write, and study at a school. I need studies and I don’t have them. I feel sad, I feel that instead of going up, I go down, because with a lack of studies everything is more difficult. Even for cleaning tasks, I need a driver’s license, I’ve been to about three or four interviews and they all ask for a driver’s license and they don’t accept me because of that, but it’s not just me, it’s with several people. Do you need a license to clean windows? For God’s sake, if they wanted to help a person in need, they wouldn’t, they wouldn’t”.*

In the case of participant P2, the lack of studies made it impossible for him to answer certain questions during the interview:

*Interviewer: “(…) Can you tell me what you mean by vulnerability and human fragility? Do you know?”; P2: “No, because I have no studies”.*

Poor literacy appeared to be an important barrier that exacerbates vulnerability and limits access to available social resources. As a second sub-theme, physical/mental frailty emerged, with greater emphasis on mental frailty. There were several participants who focused on stress, depression, anxiety, and other psychopathological manifestations.

*P6: “Wow, that’s not very easy. When I found out I had this [HIV] it wasn’t easy, you see? To accept that I had this wasn’t very easy, it didn’t really fit in my head. When I fell in the hospital, I was told what this was, now it took years and years to get it into my head. It was not easy. And even now I get anxious and stressed”.*

*P7: “My condition is difficult (…) my depression, there are things that I cannot deal with, they have already seen, that I am under enormous pressure. I went to treatments, I’ve been hospitalized a few times, they [professionals] know everything, but this is not easy. When a person is discouraged, sad, a person is forced to smoke a cigarette to kill the stress”.*

*P9: “I also had that… persecution. I also have these thoughts, that…” Interviewer: Do you think people were following you?” P9: “Yes, yes”.*

Physical and mental fragility were apparently a constant, with repercussions on people’s functioning, justifying the need for monitoring, supervision, and sometimes medical treatment.

#### 3.2.3. Strategies for Dealing with Vulnerability

Vulnerability identifies the extent and intensity of individual susceptibility to risk, while resilience is the ability to withstand and overcome risk. In this sense, participants needed to adopt strategies, such as seeking support from those who could help them. The first theme portrays the ability to ask for help/seek support, where some participants underlined its importance in dealing with vulnerability. They highlighted the importance of the professionals providing their services at InPulsar.

*P2: “I turn to people who are not my family, and these are the people who give me the most support. I came to ask for help because I wanted to change”.*

*P3:” I think I’m having it now with InPulsar than I had before. Previously, it was not easy, I was already unemployed and was denied help many times, and now, as far as I can see, I still have no reason to complain”.*

*P5: “They [InPulsar] always have their doors open to help; They have professionals in the social, health and legal areas. And they even give us psychological support”.*

*P7: “Yes, they have helped me, nobody points the finger at me, everyone helps and supports me in here, they never point the finger at me”.*

*P10: “They are really tireless”. I’m so glad I came here and ask for help in time.*

The request for help proved to be crucial as a strategy to adjust to vulnerability, namely through community institutions that help them and promote their autonomy and inclusion. This support can be carried out not only through material and financial support, but above all through emotional support.

Some participants underlined the importance of motivation and commitment to behavioural change.

*P1: “When I was living on the street, I had many moments when I thought that this was not life and that I had to change something. After all, living on the street is not a system”.*

*P6: “I am waiting to take a course, Carpentry (…). I still have time, since I didn’t learn at 18, I’m learning now at 50”.*

*P7: “I have to be a strong woman. After all, I have a son, it’s the most important thing in my life… It’s not with a social support check (189 euros) that I’m going to take my life forward. I need to find a job to give you a better life!”*

*P10: “I won’t give up, I won’t! I take methadone, it’s one of the things I want too, it’s a goal, to end methadone”.*

Another theme identified by the participants refers to not exposing others to the same risks. HIV infection alters a person’s awareness of the results of their behaviour towards sexuality. This perception transforms the sexual act and the intimate interpersonal relationship into a challenge. Some participants showed some resentment for having contracted HIV, believing that they were infected because of the insincerity of their sexual partner, whom they blame. However, they expressed concern about protecting others, despite sometimes hiding that they are HIV positive:

*P1:” Yes. I got HIV from someone who had it, but at the time he couldn’t tell me he had it and we had sex and it happened… he never told me he had HIV, I was very angry. But since then, I’ve never exchanged syringes with anyone, I’ve never had sex without a condom, always respecting others”.*

*P10: “(…) I’ve had several girls, since I have HIV it was always with a “condom” and I haven’t found the exact girl to say like that (…) Look, I have HIV”.*

Any kind of risk should be avoided, even more so when other individuals are concerned. However, issues such as sexually transmitted diseases are embarrassing, because they involve intimacy, are sometimes difficult to address, which can increase the individual’s vulnerability.

Finally, six participants mentioned ignoring the disapproving “look” of others as a coping mechanism focused on avoidance.

*P1: “I don’t know. Because I lived in Belgium for 12 years and I know what it is and for me I can ignore it”.*

*P2: “Walking around with my face uncovered and I like to pass by and they don’t point at me (…) they’re ready to look at me, but I don’t care. Move on”.*

*P3: “I never waste time on what others think about me”.*

*P5: “I don’t know, I don’t notice it! Sometimes it’s my boyfriend who says (…) but I don’t care”.*

*P6: “Now I don’t know, but before it was more difficult”.*

*P11: “When they discriminated against me, it was without me knowing, it was behind the scenes. I recognize that sometimes pretending not to see yourself may not be enough. Then we have to give it a go!”*

The “Ignore” strategy seems to help minimize suffering. However, P11 warned that prolonged maintenance of avoidance may be insufficient to deal with prejudice, and problem-focused strategies may be necessary, as these are apparently more effective.

## 4. Discussion

This qualitative study involved an in-depth exploration of the perceptions and experiences regarding vulnerability issues. A thematic analysis of the responses and their commonalities suggested several themes and sub-themes, which allow us to portray vulnerability as a concept that is both individual and universal. There is a high symbolic load associated with vulnerability, regardless of gender, age, socioeconomic stratum or cultural origin [29]. Vulnerability “is a product of situations people are living in, the so-called *vulnerable situations*. This, in turn, requires a deep and nuanced identification and analysis of elements of vulnerability that might intersect and result in differentiated degrees of vulnerability” [30] (p. 6).

According to Carmo and Guizardi [31], the concept of vulnerability is a constitutive condition of the human being, which corroborates the view, also present in our study, that vulnerability is an ontological condition that spreads. Conditions for vulnerability depend on several factors, such as biological factors, life experiences, and the environment of each individual [32,33].

Human beings cannot survive without the help of others; therefore their existence is marked by moments of greater or lesser vulnerability [34]. Our findings agree with the available evidence showing that vulnerability is typically associated with situations of social disadvantage that lead to poverty; marginalization, feelings of powerlessness/distrust, and limited or no access to resources [2,35,36]. Furthermore, lower levels of education predict higher levels of vulnerability [37]. Thus, vulnerability is caused by the lack of means and capacity of populations to protect themselves.

Our results clearly indicate that vulnerability is composed of an intrinsic dimension (that is, non-modifiable) and an extrinsic component, and therefore subject to fluctuations conditioned by the environment [38]. Both components were addressed by the participants, through the themes: an ontological condition that spreads, being alone (without a network), and being exposed to external pressure (others). UNESCO [39] stresses that the human condition, by itself, implies vulnerability, with every human being exposed to susceptibilities, whether physical or mental. Therefore, we need recognize that, at some point in life, we may not have the ability or the means to protect ourselves, as there is always the possibility of being confronted with pathologies, deficiencies, and damages from the environment or other human beings that can lead to death.

Another theme seen in the literature is life disruption caused by a lack of support and societal pressure [40], which refers to a variety of possibly overlapping factors, such as those originating from a ‘broken’ family due to substantial socioeconomic issues or other crises [41].

Regarding the barriers imposed by vulnerability, our findings show that discrimination and stigma are prevalent phenomena, experienced by some participants across life. Such prejudice made participants feel insecure and unable to affect their own future; instead, decisions were made about them and for them by individuals who didn’t comprehend their circumstances. This sense of powerlessness, evident in their perception that they have no option, was previously recorded in a Gypsy travelling community [42], where it leads to self-segregation and contributes to a general sense of mistrust. This lack of trust fosters a sense of helplessness over one’s own destiny and leads to vulnerability.

Poor physical and mental health, harmful risk-taking behaviours (e.g., drug misuse, HIV, and sexually transmitted infection) [41] and public health consequences [3,43] are frequently identified, in the literature, as barriers to and negative consequences of vulnerability. Our findings demonstrate that lack of literacy and individual frailty increased vulnerability to stressors and limited social reintegration [37]. These barriers include concerns about fitting in and becoming a well-adjusted member of society. According to Guignon [44], one can only feel authentic in a world that values unique abilities, embraces variety, provides equal opportunity, and values criticism and controversial views; a world that ensures there are no restrictions to free expression. Although some individuals feel stigmatized by society, they do not retaliate in the same way, displaying a compassionate concern for others [45].

Other elements mentioned during the interviews may be seen as a promise to overcome vulnerability and stigmatization. The ability to ask for and seek help allows the subject to overcome their difficulties, or else, provide them with tools to be able to fight their weaknesses and the threats to their balance [34]. On the other hand, the motivation/commitment to change encourages the individual to create new behaviours or stop those that are harmful to their condition [46].

From a transactional perspective, Lazarus and Folkman [47] characterize the coping construct as the individual’s cognitive and behavioural efforts to deal with internal or external challenges, evaluated as exceeding their resources. They conceptualize the categories of coping along the three dimensions of control or confrontation, avoidance, and task-oriented emotion. Control or confrontation strategies consist of proactive cognitive actions and reassessments, highlighting the themes “ability to ask for help/seek support”, “motivation/commitment to change”, and “not exposing others to the same risks”, where participants reveal a clear concern with changing their behaviour, through commitment and concern for others. Avoidance strategies, based on actions and cognitions that suggest an escape from the problem, were also identified, the main example being “ignoring the disapproving ‘look’ of others”. Finally, emotion-focused strategies were not identified by the participants, although some manifestations of stress, anxiety and depression were highlighted as interfering with their functioning and emotional regulation.

### 4.1. Study Strengths and Limitations

This study provides a timely overview of the challenges faced by vulnerable people, as seen from their perspective. This might help academics, who want to involve vulnerable people in the community and make informed suggestions on providing public services. Furthermore, by keeping a reflective research journal and peer-reviewing the developed themes and sub-themes, the research was transparent and rigorous throughout the stages of data collection and analysis.

Despite its strengths, the current study has several limitations. First, the obtained sample size was smaller than expected. Overall, participants revealed poor knowledge of vulnerability factors, due to a lack of motivation or literacy, resulting in communication issues throughout the interview process. This limitation is consistent with earlier research on contributors’ unwillingness to give their opinions owing to stigma and trauma, or lack of trust in the researchers’ intentions [48,49]. However, the small sample size was offset by the richness of our participants’ descriptions of their experiences. Second, interviews could potentially elicit unpleasant emotions and influence contributors’ health and well-being. Psychological support was offered when some emotional overburdening arose during interviews. Third, there was no data source triangulation to obtain various viewpoints (including researchers, service providers and service users). Fourth, and to protect contributors’ privacy, we did not collect individual socio-economic information. Fifth, only those who used support services were ‘heard’, which may imply that the least engaged individuals were excluded. Sixth, the study did not expressly focus on health vulnerability per se, therefore no specific health questions were addressed; however, participant beliefs and experiences of vulnerability clearly influenced their health, so this area deserves further investigation. Finally, this study outlined one way to investigate the problem of vulnerability in the Portuguese context. It made no mention of the structural reasons of vulnerability, such as limited income support, or the plethora of other systemic problems that have contributed to rising vulnerability levels. This might limit the generalizability of our findings to other contexts. Further study should take these structural and systemic aspects into account. Research may help national and local governments and organizations set specialized standards, hire workers with the required skills to address specific requirements, and inform people in suitable ways and according to the differences among groups presently depicted as vulnerable [29]. Socioecological models [14,15] provide useful frameworks to consider levels and interconnections in research. Mixed-method studies are also suggested as they can: (a) measure indicators of physical, mental, and social vulnerability; and, additionally, (b) understand the lived experience of those involved, in order to create multidisciplinary intervention programs that meet their real needs.

In addition, further research is needed to investigate practitioners’ understandings and perceptions of vulnerability, to better grasp what needs to be done to address health disparities encountered by vulnerable populations. 

### 4.2. Implications for Practice

This study enables us to reflect on the dimensions of vulnerability. Understanding the social determinants of vulnerability is necessary to achieve satisfactory care for human groups. Only by considering a population’s context and the sociocultural factors contributing to vulnerability, can we promote actions that meet and respect the collective and individual needs. Therefore, this study help can broaden our thinking and awaken our consciousness of the organizing values of health care as a social practice. This entails political–cultural–social participation to assist the health care of vulnerable human groups, as well as the adoption of attitudes and behaviours that establish and enhance health care and maintenance activities from all perspectives. Another relevant implication is the need for skills training opportunities for healthcare professionals and social workers to support them in engaging with vulnerable groups [25].

In recent decades, scholars in the Western world have paid close attention to the idea of vulnerability. Health and social care workers must explicitly apply critical views to the concept of vulnerability in order to build a more comprehensive understanding of vulnerability. By doing so, educators will be better prepared to teach about vulnerability, question the concept’s structural components and anticipate collectivist methods to address problems such as vulnerability in community contexts [50]. Torralba i Roselló [51] mentioned that the lack of a pedagogy of vulnerability has serious effects on care processes in a broad sense. Practitioners must recognize and grasp these broader societal forces, as well as ensure that services are culturally sensitive. Practitioners should also be encouraged to share information on best practices, thus contributing toward effective interventions to improve health and social outcomes [52].

## 5. Conclusions

Thematic analysis of the collected data yielded three key themes: conceptions about vulnerability; barriers imposed by vulnerability; and strategies for dealing with vulnerability. Our findings revealed that vulnerability is a very dynamic process of openness to conditions that impact individual outcomes. However, there is a conceptual gap: being vulnerable is perceived as something negative, but vulnerability also has the potential to change priorities in life for the better. This research considered the larger themes of vulnerability, helping to transform the link between how individuals might use life’s catastrophic and adverse occurrences as possibilities for positive growth, thus contributing to change our mindset concerning vulnerability. We must recognize that a person’s strength may sometimes be expressed via their vulnerability, as this allows them to reorganize and be a part of a more integrative and inclusive society.

## Figures and Tables

**Table 1:** Participants’ demographics.

Participant	Age (Years)	Sex	Educational Level
P1	43	Male	7th year (3rd cycle of basic education)
P2	56	Female	3rd year (1st cycle of basic education)
P3	24	Female	9th year (3rd cycle of basic education)
P4	41	Female	7th year (3rd cycle of basic education)
P5	25	Female	12th grade (secondary school)
P6	49	Male	Attended basic school but never finished
P7	49	Female	2nd year (1st cycle of basic education)
P8	65	Male	4th year (1st cycle of basic education)
P9	34	Male	8th year (3rd cycle of basic education)
P10	42	Male	7th year (3rd cycle of basic education)
P11	67	Male	9th year (3rd cycle of basic education)
P12	31	Female	9th year (3rd cycle of basic education)

## Data Availability

The data are available upon reasonable request.
